# Fruit Wastes as a Valuable Source of Value-Added Compounds: A Collaborative Perspective

**DOI:** 10.3390/molecules26216338

**Published:** 2021-10-20

**Authors:** Massimo Lucarini, Alessandra Durazzo, Roberta Bernini, Margherita Campo, Chiara Vita, Eliana B. Souto, Ginevra Lombardi-Boccia, Mohamed Fawzy Ramadan, Antonello Santini, Annalisa Romani

**Affiliations:** 1 CREA-Research Centre for Food and Nutrition, Via Ardeatina 546, 00178 Rome, Italy; g.lombardiboccia@crea.gov.it; 2 Department of Agriculture and Forest Sciences (DAFNE), University of Tuscia, Via San Camillo de Lellis, 01100 Viterbo, Italy; roberta.bernini@unitus.it; 3 PHYTOLAB (Pharmaceutical, Cosmetic, Food Supplement Technology and Analysis)-DiSIA, Department of Statistics, Computer Science, Applications “G. Parenti”, University of Florence, Via U. Schiff, 6-50019 Sesto Fiorentino, 50121 Florence, Italy; margherita.campo@unifi.it (M.C.); annalisa.romani@unifi.it (A.R.); 4 QuMAP-PIN S.c.r.l.-Polo Universitario “Città di Prato” Servizi didattici e scientifici per l’Università di Firenze, Piazza Giovanni Ciardi, 25-59100 Prato, Italy; chiara.vita@pin.unifi.it; 5 Department of Pharmaceutical Technology, Faculty of Pharmacy, University of Coimbra, Pólo das Ciências da Saúde, Azinhaga de Santa Comba, 3000-548 Coimbra, Portugal; souto.eliana@gmail.com; 6 CEB-Centre of Biological Engineering, University of Minho, Campus de Gualtar, 4710-057 Braga, Portugal; 7 Agricultural Biochemistry Department, Faculty of Agriculture, Zagazig University, Zagazig 44519, Egypt; hassanienmohamed@gmail.com; 8 Deanship of Scientific Research, Umm Al-Qura University, Makkah 24231, Saudi Arabia; 9 Department of Pharmacy, University of Napoli Federico II, Via D. Montesano 49, 80131 Napoli, Italy; asantini@unina.it

**Keywords:** food industry, phytochemicals, biowastes, sustainability, supplements, nutraceuticals, health, fruit wastes

## Abstract

The by-products/wastes from agro-food and in particular the fruit industry represents from one side an issue since they cannot be disposed as such for their impact on the environment but they need to be treated as a waste. However, on the other side, they are a source of bioactive healthy useful compounds which can be recovered and be the starting material for other products in the view of sustainability and a circular economy addressing the global goal of “zero waste” in the environment. An updated view of the state of art of the research on fruit wastes is here given under this perspective. The topic is defined as follows: (i) literature quantitative analysis of fruit waste/by-products, with particular regards to linkage with health; (ii) an updated view of conventional and innovative extraction procedures; (iii) high-value added compounds obtained from fruit waste and associated biological properties; (iv) fruit wastes presence and relevance in updated databases. Nowadays, the investigation of the main components and related bioactivities of fruit wastes is being continuously explored throughout integrated and multidisciplinary approaches towards the exploitation of emerging fields of application which may allow to create economic, environmental, and social value in the design of an eco-friendly approach of the fruit wastes.

## 1. Introduction

According to the United Nation’s Food and Agriculture Organization, the world production of fruits exceeded one billion tons in 2017, by generating large amounts of by-products and waste [1]. Food production and consumption generate a large quantity of processing waste biomasses, and a great part of them can be attributed to fruit waste. Fruit injuries, bruising, over-ripening during food transport and storage are reported as among the main contributors to the food waste production, and fruit wastes have been reported for nutritional and/or functional properties [2,3]. Recently, the recovery of bioactive compounds with nutritional and nutraceutical potential applications [4,5,6,7] from food industry by-products has received significant attention, mainly due to their health benefits for humans [8]. Banerjee et al. [9] reviewed bioactive products from fruit processing wastes, emphasizing green approaches to recover valuable substances. The peels, pomace, and seed fractions of fruit waste represent a good feedstock for recovery of bioactive compounds, i.e., phenolics, pectin, lipids, dietary fibers, etc. Only recently the extraction processes of active ingredients from waste are moving towards environmental certifications and LCA (Life Cycle Assessment) and LCC (Life Cycle Cost) studies, also aimed at environmental product certifications. Many of these scientific activities are funded in the context of national and European innovation and sustainability projects [10]. This paper focuses on the bioactive potential of fruit waste and by-products with possible applications in the food industry and in the health area. Particularly, the topic is declined as follows: (i) literature quantitative analysis of fruit waste/by-products, with particular emphasis on the linkage with health; (ii) an updated view of conventional and innovative extraction procedures; (iii) high-value added compounds obtained from fruit waste and associated biological properties; (iv) fruit waste presence and relevance in updated databases.

## 2. Fruit Waste and Health: Literature Quantitative Analysis

In September 2021, a search has been conducted through the Scopus Core collection database for fruit wastes research. Bibliometric data were extracted from the Scopus database (https://www.scopus.com/home.uri, accessed on 17 September 2021) using the search string: TITLE-ABS-KEY (“fruit waste *” OR “fruit by-product *” OR “fruit byproduct *”). This search strategy identified publications that mentioned the relevant words or their derivatives in the title, abstract, or keywords. As a result, the following parameters were assessed: publication year, publication count document type, authorship, institution, and Country/Region.

The “Analyze” and “Create Citation Report” functions of the Scopus web online platform were utilized for the basic analyses. A single database was selected to extract the data. Therefore, possible publications not indexed in this database are missing from this analysis.

**Figure 1:**
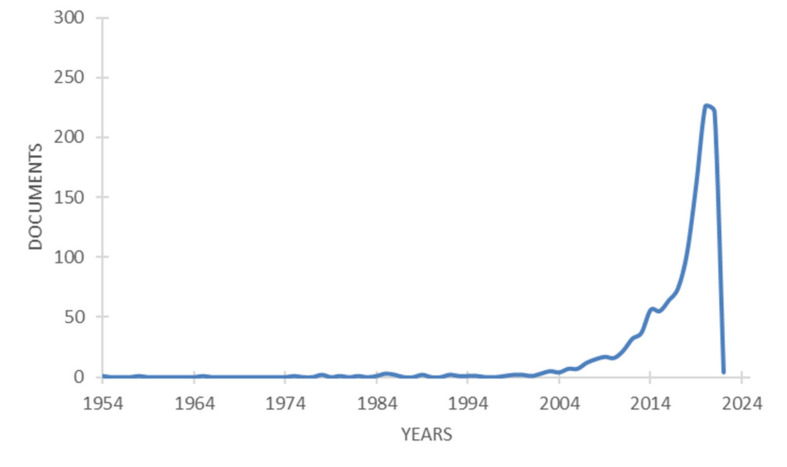
Publication trends for fruit wastes/by-products research are displayed as cumulative functions (Bibliometric data were extracted from the Scopus online database).

The search returned 1169 publications covering the period from 1954 to 2022 cited by 16,136 documents. The publications trends of fruit waste/by-products search are reported in Figure 1. 

**Figure 2:**
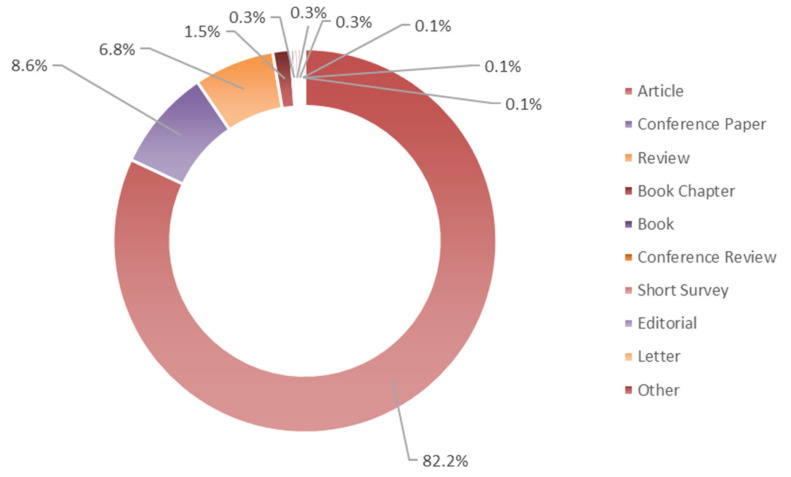
Distribution of documents by type (Bibliometric data were extracted from the Scopus online database).

The oldest publication found was published by McCready and Owens in 1954 [11] and was focused on pectin as a product of citrus waste, whereas the most recent is by Rangaraj et al. [12] which, in 2022, investigated silver–sepiolite hybrid reinforced active gelatin/date waste extract blend composite films for food packaging application. Wan Ngah et al. [13] were found to have published the most cited paper (1394 times), which summarized the research well on removing heavy metal ions from wastewater by chemically modified plant wastes as adsorbents. The types of documents related to the 1169 publications retrieved were distributed as reported in Figure 2. “Article” accounts for 82.2%, followed by “Conference paper” (8.6%), “Review” (6.8%), and “Book chapter” (1.5%). The “Book” categories that were reported were addressed on: (I) biotransformation of agricultural waste and by-products, with regards to the food, feed, fiber, fuel (4F) economy [14]; (II) recovery of high value biochemicals from waste biomass [15]; (III) seeds as functional foods and nutraceuticals [16].

One document belonging to the “Editorial” category is present and it is entitled “Sustainable processing innovations for foods” published on *Frontiers in Sustainable Food Systems* in 2020 [17].

**Figure 3:**
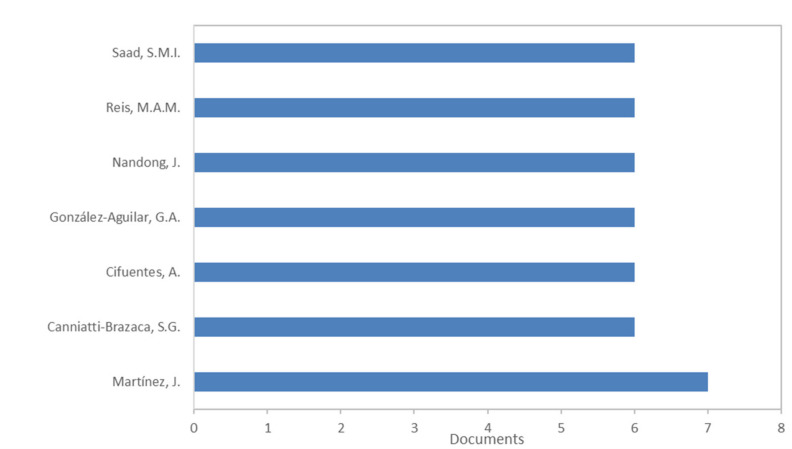
Most productive authors (Bibliometric data were extracted from the Scopus online database).

The most productive authors are indicated in Figure 3. Martínez, J. resulted the most productive Author with seven documents, whereas the others reported in Figure 2 reached six documents each. The most cited paper reported for Martínez, J. is focused on the economic evaluation of sequential multi-stage and single-stage processes of the use of supercritical fluid and pressurized liquid extractions of phytonutrients from passion fruit by-products [18]; whereas the newest paper reported for this author is addressed on application of Hansen solubility parameters strategy and inhibition of neurodegenerative enzymes for selective extraction of piceatannol from *Passiflora edulis* by-products [19].

**Figure 4:**
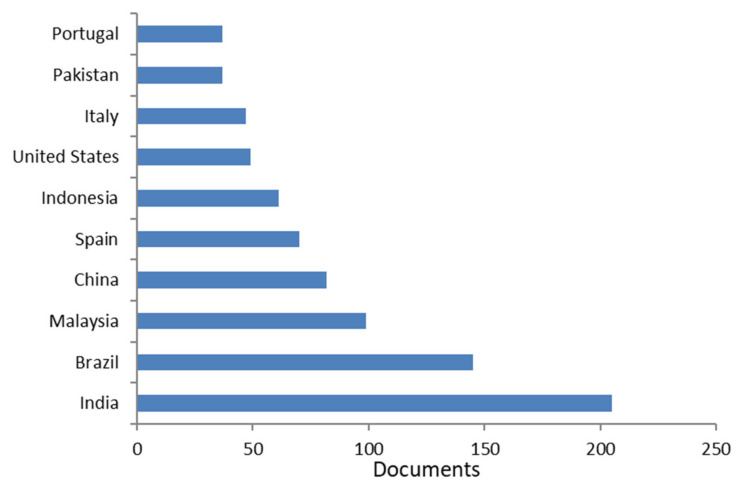
Most productive countries/territories (Bibliometric data were extracted from the Scopus online database).

**Figure 5:**
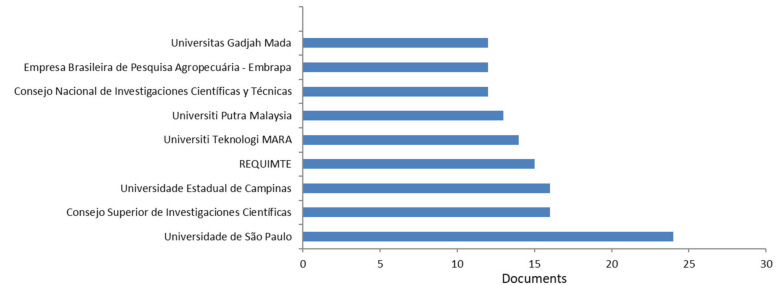
Most productive institutions (Bibliometric data were extracted from the Scopus online database).

Figure 4 and Figure 5 show the most productive countries/territories, and institutions, accordingly. Almost 37 documents have been reported for the most productive countries. India resulted the most productive country with 205 documents. The most cited work (764 times) among the 205 documents reported for India was a paper published in 2012 in the *Journal of Environmental Management*, regarding the conversion of waste products such as fruit wastes, coconut shell, scrap types, bark, and other tannin-rich materials, etc., into effective adsorbents and their application for water treatment [20].

For India, among the most recent works, beside the work of Rangaraj et al. [12] previously described, the work of Mahapatra et al. [21] described the improving the quality and shelf-life of chevon meatballs by incorporating fruit and fruit by-products. The work of Kumar et al. [22] showed spectroscopic and morphological characterization of *Nephelium lappaceum* peel extract synthesized gold nanoflowers and their catalytic activity. Another one is by Srivastava et al. [23], which summarized the technological advances for improving fungal cellulase production from fruit wastes for bioenergy application. Particular emphasis was also given to the various bottlenecks and feasible approaches such as the use of nanomaterials, co-culture, molecular techniques, genetic engineering, and cost economy analysis to decrease the overall cost. The paper of Murphin Kumar et al. [24] is also interesting, as it addressed the development of tropical fruit waste-derived mesoporous rock-like Fe_2_O_3_/C composite fabricated with amphiphilic surfactant-templating approach showing massive potential for high-tech application [24].

Among the 145 documents reported for Brazil, the most cited work (305 times) [25] aimed to test yellow passion fruit (*Passiflora edulis Sims*. f. *flavicarpa Degener*) (YPFW) a powdered solid waste, as a biosorbent for the removal of a cationic dye, methylene blue (MB), from aqueous solutions. Taking into account the analysis of the normal distribution of the residuals (difference of q_measured_-q_model_), the data were best fitted to Sips isotherm model. The maximum amount of MB adsorbed on YPFW biosorbent was 44.70 mg g^−1^ [25]. 

Its most recent work was published by Silva et al. [26] on *Food Chemistry* in 2021 on how pomegranate (*Punica granatum*) peel fractions by supercritical CO_2_ increase oxidative and color stability of bluefish (*Pomatomus saltatrix*) patties treated by UV-C irradiation

The most cited work for Malaysia was the review of Wan Ngah et al. [13] previously described. On the other hand, the most recent work of Ling et al. [27] was focused on natural deep eutectic solvent composed of choline chloride, and ascorbic acid (CHCL/AA NADES) was formulated for enhancing the solubility and antioxidant properties of antioxidant extracts from fruit wastes of *Mangifera pajang*.

Then, work by Solangi et al. [28], published in the *Journal of Hazardous Materials* in 2021 also reviewed the development of fruit waste derived bio-adsorbents for wastewater treatment.

The study of Tan et al. [29] addressed the utilization of tropical fruit wastes as an organic nutrient source for the cultivation of *Chlorella vulgaris* and *Haematococcus pluvialis*.

The most productive institutions were the Universidade de São Paulo with 24 documents. The most cited work for Universidade de São Paulo is published by Do Espírito Santo et al. [30] in the *International Journal of Food Microbiology* in 2012 on the utilization of fibers from fruit by-products for enhancing probiotic viability and fatty acid profile and increase CLA content in yoghurts. Their newest work was focused on extraction of phenolic compounds from acerola by-products using chitosan solution, encapsulation and application in extending the shelf-life of guava [31]. 

The most cited work for Consejo Superior de Investigaciones Científicas is by Fernández-Gómez et al. [32] on the utilization of continuous-feeding vermicomposting as a recycling management method to revalue tomato fruit wastes from greenhouse crops. 

The work of Castro et al. [33] is the most cited for Universidade Estadual de Campinas and studied the preconcentration procedure of metal ions with a fruit waste, through a case study of banana peel applied to the solid phase extraction of copper and lead from river water. For REQUIMTE, the most cited work described combined strategies to boost polyhydroxyalkanoate production from fruit waste in a three-stage pilot plant [34]. The most cited work (24 times) for the Universiti Teknologi MARA is the work of Daud et al. [35] on the evaluation of the antioxidant potential of *Artocarpus heterophyllus* L. J33 variety fruit waste from different extraction methods and identification of phenolic constituents by LC-MS. The maceration technique yielded extracts with the strongest antioxidant activities which can be correlated with the highest total phenol and flavonoid content values. The liquid chromatograph-mass spectrometry (LC/MS) analyses identified two phenolic acids, protocatechuic acid, which is one of the major metabolites of antioxidant polyphenols present in green tea, and chlorogenic acid as the major constituents responsible for the antioxidant activity of the active extracts. 

Addressing the search towards the relationship between fruits waste/by-products, and health, the search has been carried out using the string: TITLE-ABS-KEY (“fruit waste *” OR “fruit by-product *” OR “fruit byproduct *” AND “Health *”). For further bibliometric analyses and additional processing, the “full records and cited references” were exported to VOSviewer software (version 1.6.16, www.vosviewer.com, accessed on 19 September 2021). The VOSviewer software (v.1.6.16, 2020) [36,37,38] analyzes the terms/words used in the titles and abstracts of publications by breaking down the paragraphs into words and phrases, linking them with the citation data of the publications, and visualizes the results in the form of a bubble map by using a term map with default settings. To simplify the bubble map, words/terms that appeared in at least five of the publications were analyzed and visualized. Of the 1756 keywords, 59 met the selected threshold, and four of them were manually excluded. 

The search returned 123 publications covering the period from 1975 to 2021. The most recent work is by Millan-Linares et al. [39], which investigated the use of pectins and olive pectins in an application area ranging from biotechnology to human health. Other studies were on: (i) effects of foliar and soil-applied liquid organic fertilizers on the growth of *basella alba* l. and *centella asiatica* l [40]; (ii) advantageous nutritional and functional properties of Baru (Dipteryx alata Vog.) fruit as an option of nut and pulp [41]; (iii) development of frozen pulps and powders from carrot and tomato by-products [42]; (iv) dietary fiber and prebiotic compounds in fruits and vegetables food waste [43]; (v) lipophilic extracts isolated from European cranberry bush (*Viburnum opulus*) and sea buckthorn (*Hippophae rhamnoides*) berry pomace by supercritical CO_2_ as promising bioactive ingredients for foods and nutraceuticals [44].

The most cited “Article” is published by Larrauri [45] on *Trends in Food Science and Technology* in 1999, which focused on new approaches in the preparation of high dietary fiber powders from fruit by-products, whereas the most cited Review (330 times) is published in *Food Research International* in 2011 by Ayala-Zavala et al. [46] on the agro-industrial potential of exotic fruit byproducts as a source of food additives. 

Among 123 documents, focusing on “Medicine” Subject Area, the most cited paper is a study of Batista et al. [47] on beneficial effects of consumption of acerola, cashew or guava processing by-products on intestinal health and lipid metabolism in dyslipidemic female Wistar rats, whereas the most recent paper is published by Hasted et al. [48] on the immunostimulatory potential of fruits and their extracts in poultry

**Figure 6:**
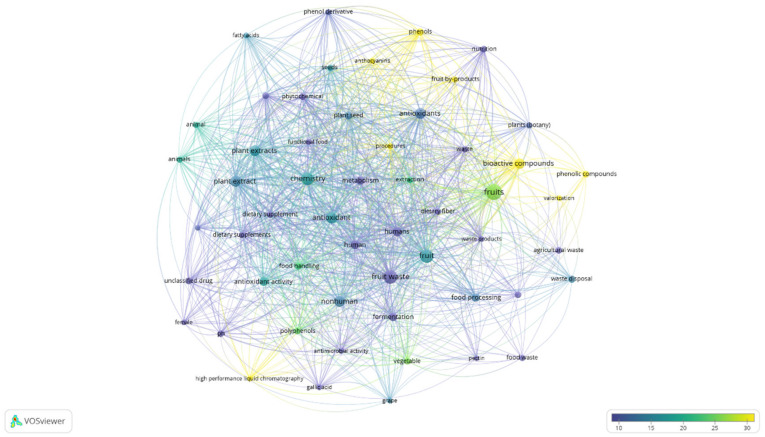
Term map for fruit waste/by-product and health research. Bubble size represents the number of publications. Bubble color represents the citations per publication (CPP). Two bubbles are closer to each other if the terms co-appeared more frequently (Bibliometric data were extracted from the Scopus online database and elaborated by VOSviewer software).

A total of 55 terms were derived from the quantitative literature research on publications, and they are visualized as a term map shown in the Figure 6. Among the top-recurring terms for fruit waste/by-product and health (Table 1), are: chemistry, bioactive compounds, phytochemicals, antioxidant/s, antioxidant activity, plant extract/s, by highlighting as the combined and concerted actions of biologically active components can be viewed as a potential indicator of health related to plant extract such as from fruit wastes. At the same time, the importance of innovative technologies and procedures, to obtain a functional bioactive-component rich extracts, is revealed.

## 3. Conventional and Innovative Extraction Procedures: An Updated Shot

The specific extraction methodologies for each different raw material on an industrial scale are selected on the basis of their physico-chemical characteristics, the natural compounds and active molecules to recover, and the yields in targeted compounds. However, more important factors may be needed to ensure the industrial process is suitable for different matrices, the other processes are integrated in the whole biorefinery plant, and for environmental and economic sustainability. Therefore, the choice of a particular extraction technique, for one or more classes of bioactive compounds from waste, depends on a compromise between the above-described factors. The recovery and reuse of waste and by-products are performed according to a Circular Economy model that nowadays includes the concepts of biorefinery and industrial symbiosis. In this context, the technologies and procedures are aimed at increasing the sustainability, avoiding or minimizing the use of organic solvents, as well as any other impact on the environment. Moreover, on a lab-scale, new procedures for chemical characterization of vegetal waste and by-products, including the preparation of the sample, have been recently developed to replace the conventional methods of solvent extraction based on the use of organic solvents, which may cause environment and health issues, preferring the green chemistry approach and sustainable methodologies [49,50,51,52]. The most widespread innovative techniques, in addition or in place of simple hydroalcoholic extraction, are enzymatic treatment, ultrasound-assisted extraction, microwave-assisted extraction, supercritical and subcritical fluid extraction, and other techniques such as pulsed electric field extraction, water extraction, and pressurized liquid extraction. Although, it must be taken into account that most of natural molecules are not stable to thermal treatments and should be avoided as much as possible. The extraction process may be generally preceded by further online steps for the preparation of the raw material, such as separation of different vegetal tissues, cleaning or disinfection, drying and/or milling, and followed by purification steps performed by membrane separation techniques, vacuum concentration and spray drying and/or granulation process to obtain dried extracts, which are more stable and better manageable than concentrated solutions [51,52,53,54,55,56,57,58].

Concerning fruit waste and their by-products, the enzymatic treatment is used to extract natural compounds such as pectins or polyphenols, and is applied as such or combined with conventional extraction techniques, e.g., hydroalcoholic extraction, or with non-conventional techniques, to increase yields of bioactive compounds and minimizing extraction times and temperatures [50]. At a laboratory scale, the use of enzymes is often associated with the presence of hydroalcoholic solutions and/or suitable buffers, being crucial for yields and composition of the extracts not only the specific enzyme or enzymes mixture, but also pH, temperature, extraction time, and solid/solvent ratio [59,60,61]. The enzymatic extraction, up-scaled to an industrial process, is very effective with respect to the conventional solvent extraction, especially for “hard” matrices (e.g., peel, seeds) and non-soluble compounds such as polymerized polyphenols (e.g., proanthocyanidins) or heavy molecular weight pectins, allowing for lower extraction times and temperatures. Studies on the industrial extraction efficiency of different techniques in winemaking by-products, in particular grape pomace, seeds and peel, confirmed that enzymatic treatment, eventually combined with hydroalcoholic extraction, strongly increased the yields of non-soluble or scarcely soluble compounds such as monomeric phenolic acids and procyanidin dimers A and B [62,63]. In the literature, industrial enzyme-assisted extraction is reported as a suitable technique in particular for obtaining pectins and polyphenols from waste and by-products, - namely pomace, peel, and seeds- from fruits like citrus, apple, berries, passion fruit, grape, kiwifruit, and other kinds of vegetable matrices (e.g., wood, bulbs, and roots). The applied temperatures are on the range from 30 °C to 50 °C, and up to a temperature of 85 °C, with a time extraction of 0.5 h up to 72 h. The yields in targeted compounds range from 2% (passion fruit peel) to 26% (lime peel) [64,65,66,67,68,69]. Moreover, the use of specific enzymes or mixtures of enzymes and the application of different process parameters (temperature, time, concentration, and pH) allows for the standardization of the obtained compounds, concerning not only the yields but also the degree of esterification for pectins and the molecular weight for polymeric tannins [50]. Other non-conventional extraction techniques are often associated with enzymatic treatment, such as ultrasound-assisted extraction, supercritical and subcritical fluid extraction, microwave-assisted extraction, or pressurized fluids extraction. They may take place at different levels in the industrial process further increasing both the process yields and its sustainability by decreasing the energy and solvents consumption. As an example, a laboratory scale procedure has been optimized by testing ultrasounds as a pre-treatment followed by extraction with a mixture of lytic enzymes (viscozyme) for the extraction of polyphenols from pomegranate peels. 

The final extract showed a total polyphenols content of 19.77 mg GAE/g, total flavonoids content 17.97 mg QE/g and 74.213% antiradical activity measured by DPPH (2,2-diphenyl-1-picrylhydrazyl) in vitro assay [70]. Moreover, ultrasounds were applied simultaneously with specific enzymes treatment for extracting anthocyanins from grape [71], whereas the use of supercritical CO_2_ is reported after enzymatic pre-treatment for the extraction of phenolics from pomegranate peel [72]. 

Ultrasound-assisted extraction is a widespread technique for both laboratory and industrial extraction of bioactive compounds such as polyphenols, carbohydrates, pectins, fiber, and organic acids from fruit matrices; the extraction yields are increased with respect to those obtained by conventional solvent extraction, as ultrasound waves can induce cavitation with erosion of the plant tissues, fragmentation of the cells in the sample, and release of bioactive compounds. Time, temperature, and pH are crucial parameters for the extraction process, in addition to the frequency and power of the ultrasounds. The frequencies used for the extraction of vegetal matrices varied from 20 kHz to 100 kHz, with power source generally from 140 W to 800 W, varying based on the raw material and the other conditions applied [51,57,73,74,75]. In the literature, ultrasound-assisted extraction has been reported for obtaining pectins from grape pomace, peel of pomegranate, grapefruit, passion fruit, banana, mango, and orange, with frequencies of about 20 kHz, and power sources from 130 W to 200 W, sonication times from 4 min to 60 min, temperatures between 35 °C and 80 °C, and, in some cases, an acidity correction up to a value of pH 1 to 2 [49,76,77,78,79,80,81,82,83]. Extraction of dietary fiber from papaya peel and apple pomace, organic acids such as tartaric and malic from grape waste and anthocyanins from grape peel is also reported with similar process parameters, and different pH values (NaOH solution or acidified water) [84,85]. Ultrasound-assisted extraction was assessed for the isolation of polyphenols from mango peel and compared to the conventional maceration technique with different solvents, leading higher polyphenols yields measured by both High Performance Chromatography and in vitro antioxidant and radical scavenging capacity assays [86]. For waste and by-products of grape processing, ultrasound-assisted extraction of polyphenols, flavonoids in particular, showed higher yields with respect to the conventional solid–liquid extraction or, combined with the solvent extraction, highly increased the efficiency of the process [52].

Ultrasounds application increased the yields of extraction of carotenoids, in particular the β-carotene from peels of mandarin (*Citrus reticulata*) and orange [87,88]. In the design and optimization of ultrasound extraction processes it must be considered that not all natural compounds are stable under the conditions usually applied. In a recent study by Qiao et al. [89], the stability of the main flavonoids from *citrus* fruits was assessed by ultrasound treatment in different conditions. Among them, only quercetin significantly degraded as a result of oxidation, addition, polymerization, and decomposition reactions, while its 3-O-rhamnoside (quercitrin), eriocitrin, narirutin, neohesperidin, eridictyol, didymin, naringenin, luteolin, sinensetin, nobiletin, tangeretin, naringin, and hesperidin remained stable. The main factors affecting flavonoids degradation reactions were the type of solvent and temperature, liquid height, ultrasounds intensity, pulse length, and duty cycle which influenced the rate of degradation but not the nature of the degradation reactions [89].

Additionally, microwave-assisted extraction is considered an efficient method, allowing for short extraction times, high extraction rates, selectivity and yields, and low cost, in some cases resulting more convenient than the ultrasound extraction approach [90]. The extraction principle is based on the ability of microwaves, usually at a constant frequency of 2450 MHz, of penetrating biomaterials producing a strong heating effect on the moisture inside the cells. The sudden evaporation of moisture generates high pressures on the cell wall from inside causing its rupture and the release of bioactive molecules. The heating efficiency of different solvents used to impregnate plant matrix can further improve the process yields, as microwave-assisted extraction is usually combined with traditional solvent extraction. The main parameters influencing the quality and the yields of bioactive metabolites extracted are: microwave power (ranging from 150 W to 500 W), pH, time of irradiation (in the range from 60 s to180 s), choice of the solvent and its polarity and dielectric constant; solid–liquid ratio (10 g/mL to 30 g/mL). The pre-treatments of the raw material such as drying and grinding can affect not only the contact surface with the solvent but also the moisture content [91,92,93]. Concerning the high temperatures generated inside the tissues of the raw material, the short time used in the microwave irradiation avoids the degradation of the active compounds [94].

Moreover, shortening the extraction times allows to preserve labile compounds such as the anthocyanins. This technique is reported for the extraction of polyphenols, pectins, polysaccharides from various plant matrices including fruit waste. Polyphenols were extracted with high yields from apple and grape pomace [90,95]; pectins from peels of mango, papaya, grapefruit, and other citrus fruits; it has also been used to extract pectins from jackfruit peel in combination with ultrasound treatment [96]. In particular, microwave assisted extraction was tested for obtaining pectic polysaccharide from mango peel, finding that the optimum extraction conditions were: microwave power of 413 W, pH of 2.7, time of 134 s, and solid–liquid ratio of 1:18 g/mL. These conditions provided the highest pectin yield, 28.86% [93]. Recently, microwave-assisted hydrodistillation has been used to obtain essential oils from bergamot albedo and lemon peel, and other citrus fruits peel waste [88,97,98]. The scale-up limitations from laboratory to a larger scale process were studied in particular for grape waste, for which this technique is particularly widespread. The main issue concerns low penetration depth, which avoids a homogeneous irradiation for large amounts of raw material without the ability to vary the microwave frequency accordingly, and would imply the counterproductive reduction of the thermal effect. A possible solution is the use of a short microwave pre-treatment followed by conventional solvent extraction. In the experiments described by Álvarez et al., [95] for grape waste extraction, microwave pre-treatment allowed to improve yield and polyphenol richness at the same time and the effect was more pronounced on polyphenols than on other compounds, such as sugar and fibers, leading to the maximum phenolics yield and richness [95,99]. However, compared to other techniques, microwave-assisted extraction may require additional filtration or centrifugation steps to remove the solid residue. Moreover, the efficiency of microwaves is very limited in extracting non-polar or volatile compounds due to the characteristics of the solvents involved in the process [91]. 

Recently, pulsed electric fields extraction has been tested and optimized for extraction of juice and bioactive compounds from fruits and their waste products as an efficient alternative to conventional methods, as a pre-treatment or in combined extraction techniques. The permeability of cell membranes is increased by applying short voltage pulses (from few μs up to 1 ms) of moderate intensity electric fields with low energy (0.5–10 KV/cm; 1–10 KJ/Kg), which enhances the release of juice and bioactive metabolites in the matrix. This technique was applied as a pre-treatment before the extraction of juice from blueberry fruits by pressing, and before solvent extraction of bioactive phenolics from the press cake. The pretreatment by pulsed electric fields significantly increased both the juice yield (+28%) and its total phenolic content (+43%), total anthocyanin content (+60%), and antioxidant activity (+31%). Additionally, the press cake extract had higher amounts of total phenolics (+63%), total athocyanins (+78%), and antioxidant activity (+65%) compared to the untreated sample. These results suggest potential industrial applications of pulsed electric fields extraction in sustainable processes, for the industrial processing of berry fruits and their waste or by-products [100]. 

Hot pressurized liquid extraction is a very widespread methodology of extraction, used for large-scale industrial applications for recovering bioactive compounds from fruits and their by-products. With respect to conventional liquid–solid extraction, this liquid–solid extraction technique is performed by applying high temperatures (from 50 °C to 200 °C) and pressures (in the range from 3 MPa to 20 MPa), which allows for shorter extraction times and the use of sustainable solvents such as water or ethanol/water mixtures, with lower costs. One of the limitations is given by the low selectivity of the methodology, that is not capable of separating compounds from similar classes, so that a purification phase, e.g., by solid adsorbents, is often needed. Da Silva et al. [101] recently developed an on-line method of extraction and fractionation for phenolic compounds from apple pomace, by coupling pressurized liquid extraction and solid-phase extraction for the separation, also replacing methanol with more sustainable solvents such as ethanol [101]. Another limitation is that the high temperatures applied often induce the degradation of polyphenols and other bioactive metabolites, lowering the yields of targeted compounds and generating the presence of degradation derivatives in the extracts. Thus, further purification and concentration steps are needed [101,102]. This problem can be partially solved by using low percentages of ethanol instead of pure water, which allows for keeping sufficient extraction yields, or increasing them, while lowering the extraction temperatures. In a recent study, 15% ethanol was used for the extraction of polyphenolic compounds from grape pomace. The polyphenols recovery (~24 mg Gallic Acid Equivalents g^−1^) did not change substantially after reducing the extraction temperature from 130 to 90 °C; moreover, lower amounts of both reducing sugars and degradation compounds were found in the extract [102].

In the last years, also supercritical-fluid extraction has played an important role in exploiting plant waste for obtaining bioactive principles such as oils, fatty acids, vitamins, antioxidants, dyes, biopolymers in an industrial, and/or biorefinery perspective. A supercritical fluid is obtained by keeping it at pressures and temperatures above the critical point. This allows to easily tune density and transport properties between the gas-like to liquid-like state by slightly changing pressure and temperature, to make more selective the extraction of compounds with different polarities [58,103]. The choice of the supercritical fluid is another crucial factor for the selectivity and yield of the extraction, but it is addressed also according to its cost, being this kind of extraction particularly expensive in general. The low cost of CO_2_, its characteristics of non-toxicity and non-flammability, and its easy availability with respect to other solvents make it one of the most used fluids at an industrial scale for the extraction of non-polar compounds such as oils, hydrocarbons, and essential oils. Polar bioactive principles such as phenolics, alkaloids, and glycosidic compounds cannot be extracted with carbon dioxide, CO_2_, hence a different solvent must be used such as Freon-22, nitrous oxide and hexane. However, the addition to the supercritical CO_2_ of polar co-solvents (modifiers) such as methanol, ethanol, acetonitrile, acetone, water, ethyl ether, dichloromethane, or mixtures of solvents is often preferred to increase and adjust the solubility of polar compounds. Among them, ethanol is not the most effective but it is the best compromise considering its miscibility with CO_2_, environmental sustainability, and lower toxicity [84]. Considering an industrial and biorefinery context, this technique is very effective due to the versatility obtainable by intervening with slight modifications of pressures and temperatures and the use of modifiers; the high yields and extraction rates due to diffusivity of the supercritical fluid; the low temperatures applied (around 30 °C for CO_2_); moreover, a fractionation of the extracted compounds is possible by controlling the fluid density and temperature, without implementing the whole process with further steps. Additionally, the concentration and recovery of the extracted active principles are possible without further processes, only by reducing the density of the supercritical fluid that is more volatile even than organic bioactive volatile compounds. On the other hand, together with these advantages, some critical points are given by the characteristics of the raw material: the water content of fresh fruits can freeze and cause mechanical problems; polar compounds may tend to remain in the aqueous phase and lower the extraction yield. The particle size of the raw material can affect the yield in different ways, so that the optimum size must be assessed for each different process [91]. Until now, these last factors made supercritical-fluid extraction applications restricted to very specialized fields such as essential oil extraction, coffee decaffeination or academic research, but the inclusion of this process in an integrated platform or biorefinery could change its possibilities of application. In a recent article by Villacìs-Chiriboga et al. [103], the valorization of by-products from tropical fruits is reviewed in a biorefinery perspective. Together with its advantages, some critical points are highlighted, such as the high investment cost, that in several countries can heavily affect its feasibility, especially considering the areas where most tropical fruits are produced. However, the high content of these waste matrices in bioactive compounds are well extractable by supercritical CO_2_ which could make their recovery and reuse more convenient than their disposal, also considering the advantages of this technique. Moreover, it must be taken into account that on industrial scale supercritical-fluid extraction plant, the main factor affecting the costs is given by the raw material, whereas the cost related to labor and the variable costs, are lower due to the high level of mechanization and the possibility of avoiding purification and concentration steps which would require suitable equipment and energy consumption. The possibility of recycling CO_2_ on industrial scale systems contributes to lowering the process cost [18,104].

In a Circular Economy and sustainable technology context, biorefineries are considered the most complete way for the creation of an industry based on green products derived from natural materials, able to recover and enhance separately the different chemical components of biomass, providing for a complete exploitation of by-products and their application in industrial sectors that may be different from such of primary product.

Here the extraction process, based on one single technology or a combination of different technologies as described above, are integrated with other steps such as: pre-treatment of the vegetal biomass (drying, milling, etc.); refining of extracts via fractionation, purification and concentration, spray-drying to obtain powdered extracts; cogeneration of energy, to obtain a wide range of bio-based products, such as nutraceuticals and functional ingredients, food and feed, chemicals, materials, fuels, power, and heat [55,56,87,88,89,90,91,92,93,94,95,96,97,98,99,100,101,102,103,104,105,106]. Membrane separation, an environmentally friendly physical separation method based on molecular weight, is the most diffused green technology for polyphenols-rich extracts fractionation, purification, and concentration, but it is suitable also for the selective extraction of bioactive compounds; it is used also for the treatment of industrial wastewater. This process generally includes several filtration steps by using semi-permeable membranes with different pore size, typically microfiltration, ultrafiltration, nanofiltration, and reverse osmosis. The pore sizes decrease from 10 to 2 µm (microfiltration) to 10–3 µm (nanofiltration) and separation is driven by pressures from 100 to 600–1000 KPa; the final reverse osmosis step is aimed at purifying and possibly recovering wastewater, that can be reused as the extraction solvent. This kind of technology avoids the use of organic solvents and is therefore preferred to other methodologies such as resin fractionation for sustainable processes. Moreover, integrated in industrial biorefinery processes for recovering bioactive compounds from fruit waste, it allows for low energy consumption, high efficiency, mild operation conditions [52].

Recently, industrial sustainable platforms for the recovery and exploitation of fruit waste were implemented, where the raw material is extracted to obtain bioactive principles, but it can be transformed into a new commercial product even as such, after drying and micronization treatments. In this latter case, the powder obtained can be used as a functional ingredient to be added to food and/or feed; for nutritional food (peel flour, dilatory fiber, jelly, bakery products). Biorefinery extraction and purification processes allow for obtaining new products and semi-finished products from fruit waste such as biomaterials (dyes for foods and textiles, bio-polymers, agronomic products, biochar), biochemicals (polyphenols and phenols, polysaccharides, pectins, proteins, enzymes, organic acids, vitamins, fatty acids, essential oils) and bioenergy products such as exhausted vegetal residues after extraction, coke, ethanol or methanol, bio-oils [55,56,106,107]. In Table 2, a brief summary of what reported above is shown with reference to the main fruit waste and by-products available in the scientific literature and/or exploited in industrial processes. 

Studies are available in the literature comparing the efficiencies of different techniques applied to specific kinds of fruits or vegetal waste and bioactive compounds in terms of yields and environmental sustainability. An interesting example is given by the extraction of pectins, complex heteropolysaccharides containing homogalacturonan, rhamnogalacturonan I, rhamnogalacturonan II in different amounts according to the different vegetal species. Several extraction methods were tested, aiming to obtain final products selectively enriched in one of these components. In fact, while pectin-rich extracts are traditionally used in food products for their gelling properties, recently innovative and more specific applications were assessed for enriched extracts in one or another of the reported components in different sectors [113]. The industrial selective extraction became commercially interesting since the scientific demonstration of different functional and biological properties for the different components, in particular rhamnogalacturonan I, that were found to be suitable for the formulation of nutraceutical or pharmaceutical products due to its properties such as immunomodulation activity [114,115]. Recently, conventional and non-conventional extraction technologies were tested to assess which treatments are able to enhance the selective extraction and modulate the composition of the extracts, increasing the extraction yield of the individual compounds, taking also into account that different fruit waste may contain pectins with different characteristics [113]. Concerning the yields in total pectin, conventional extraction methods based on chemical additives, and high temperatures to induce their release from cell walls allow for pectin yields from 0.6% to 25.6%, molecular structures are scarcely preserved due to the conditions applied during the extraction process. In particular, acid treatment can produce higher yields in pectins and extracts enriched in homogalacturonan, whereas alkali extraction is more suitable to preserve rhamnogalacturonan I, but with lower yields in pectins. Among non-conventional technologies, non-thermal methods such as ultrasounds extraction, dielectric barrier discharge plasma extraction, and enzyme-assisted extraction are better to obtain pectin rich in rhamnogalacturonan I (38.3% to 90.3%); whereas thermal methods like high pressure extraction, microwave-assisted extraction and subcritical water extraction allow for obtaining pectin with 20.7% to 85.7% Galactose A, the main component of homogalacturonan [113]. Thus, the choice of the extraction technology and the conditions to apply depends on the characteristics needed for the extract, according to the destination of the final product. Regardless, at an industrial and biorefinery level, the choice of the best extraction methodology for one specific raw material or targeted class of bioactive compounds needs to take into account not only the yields and characteristics of the extracts, but also other factors concerning the whole productive process and the economic and social context of the area where the plant is located (investment cost; other processes to be integrated in the biorefinery plant; waste availability and transport; solvent, energy, and maintenance costs; availability and cost of labor etc.). In this section we have focused on the main processes and technologies already industrially applied or in an industrial application perspective, therefore it was possible to only partially compare the different techniques applied for each analyte. Moreover, as can be seen, in some cases the same technique applied to the same raw material, but with different conditions and parameters, may yield extracts with substantially different compositions. This type of considerations needs a separate and in-depth discussion, taking into account all the above-mentioned variables.

This kind of sustainable integrated method to exploit agro-industrial edible and non-edible waste and by-products is suitable not only for fruit, but also for other vegetal species of economic and environmental interest such as olive tree, artichoke, sweet chestnut, etc. [10,55,56,106,116].

## 4. High-Value Added Compounds from Fruit Waste and Biological Properties

Fruit waste and by-products from their manufacturing represent a precious source of high added-value compounds (e.g., primary and secondary metabolites) to be extracted and valorized in the context of circular economy and environmental impact reduction [117]. 

Primary metabolites as carbohydrates, fats, proteins, and nucleic acids, are produced in large quantities from plants and are responsible for their vital functions as growth, development, and reproduction. Polysaccharides as cellulose and pectins, can be extracted from fruit waste and used for a variety of industrial applications. Cellulose is a linear polysaccharide polymer consisting of several hundred to many thousands of D-glucose monosaccharide units linked by β-1,4-bonds. The different rows are linked together by hydrogen bonds giving rise to a rigid and compact structure. For these properties, cellulose represents the most important structural component of the plants cell walls.

Corn husk and grape stalk [118], pomegranate peel, marc of strawberry-tree fruit [119], chestnut shell [120], banana [121], and orange peel [122] are sources of cellulose. Being a natural, biodegradable, and versatile polymer, cellulose can be used for a variety of several food and nonfood applications as in textile, paper, wood, pharmaceutical, and industrial chemistry. In addition, it is useful for the production of micro and nanocellulose for materials sciences and biomedical applications, specifically as carriers in drug delivery [123,124,125].

Pectins are another group carbohydrate macromolecules found in cell walls. The main monomer is D-galacturonic acid and each unit is linked to the other by an α-(1,4) bond. These polysaccharides are water soluble and show excellent thickening, gelling properties, hydration capacity, and swelling. For these peculiar properties, they are used as an additive or ingredient for food in the preparation of jellies, jams, and marmalades but also in the pharmaceutical industry [126]. The richest source of pectins are citrus fruits peel, in particular lemon peel [127,128] but also apple pomace [129] and mango peel [130]. Pectins exhibit several health benefits in humans. Being non-digestible carbohydrates in the human gut, they play important nutritional roles as dietary fiber such as intestinal transit modulation and gut microflora growth substrate. They reduce low-density lipoprotein (LDL) levels and cholesterol levels; they show antioxidant capacity and have been attributed also an important role in preventing cancer [131].

Secondary metabolites are a large variety of compounds produced in small quantities from plants for which they play a wide number of activities. For instance, they act as defense agents against predators and insects, promoters of pollination, pigments of flowers, and fruits [132]. These molecules are present in all plant organs but their qualitative and quantitative profile depends on biotic and abiotic factors. They show a wide number of biological activities and a variety of beneficial properties for human health including antioxidant, anti-inflammatory, anticancer, antifungal, and antibacterial activities [133]. For these relevant biological properties and their potential use in industrial applications, these compounds are very attractive to be used in food supplements, and as antioxidant or anti-microbial agents. Therefore, their recovery from fruit waste and reuse represents an excellent example of circular economy applied to the agro-industrial sector [134]. 

**Figure 7:**
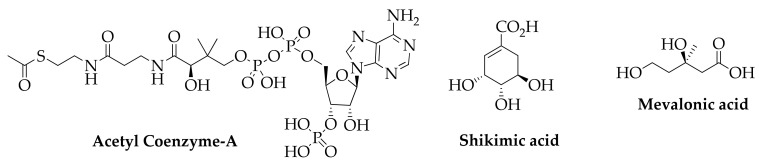
Building blocks for the biosynthesis of secondary metabolites.

Secondary metabolites are synthetized from plants mainly from the three building blocks depicted in Figure 7, namely acetyl Coenzyme A, shikimic acid, and mevalonic acid through complex enzymatic reactions which constitute the acetate, shikimate, and mevalonate pathways [135].

Fatty acids and polyketides as saturated and unsaturated fatty acids, acetylenic acids, prostaglandins, tromboxanes, and leukotrienes are synthetized from combinations of acetate units. Aromatic amino acids, polyphenols, phenylpropenes, and coumarins derive from the shikimate pathway, while terpenes and steroids derive from the mevalonate pathway. Other important secondary metabolites produced from plants are alkaloids obtained starting from amino acids such as L-ornithine, L-lysine, nicotinic acid, L-tyrosine, L-tryptophan, anthanilic acid, and L-histidine.

**Figure 8:**
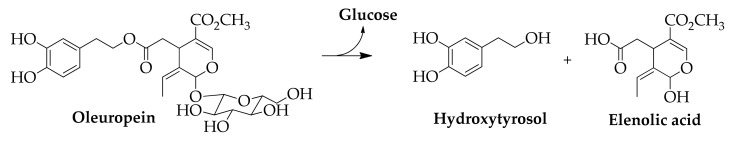
From oleuropein to hydroxytyrosol.

Polyphenols are the largest number of compounds distributed in the plants. Chemically, they are characterized from one or more aromatic ring having at least a hydroxyl group. They include both structurally simple compounds as benzoic acids, cinnamic acids, flavonoids, stilbenes, flavolignans, isoflavonoids, and complex polymers such as lignins and tannins [136,137]. Polyphenols are present in all plant organs (seeds, leaves, roots and stems). Therefore, waste deriving from fruits consumption and processing are rich of these valuable compounds. Significant sources of polyphenols are olive oil and wine wastes and by-products from their manufacturing processes. Oleuropein (Figure 8) is a secoiridoid found in olive leaves; every year after tree pruning and olive oil production about 25 kg per year of leaves were produced. It is also found in olives and is responsible for the bitter taste of fruit and olive oil. During maturation and oil processing, it is hydrolyzed by enzymes as β-glucosidase and esterase producing glucose, hydroxytyrosol, and elenolic acid [138]. The hydrolysis is observed also in presence of other factors as air, light, acid, base, oxidant agents, metal ions and high temperatures [139]. Olive leaf extract has shown antioxidant, anti-inflammatory, anti-thrombotic, antihypertensive, and antimicrobial activity [106,140,141].

Hydroxytyrosol is a catecholic compound found in olives and, after olive oil production, in olive pomace and wastewaters from which it can be recovered by sustainable technologies [142]. It shows antioxidant [143], anticancer and antimicrobial activities [144,145,146]. Both oleuropein and hydroxytyrosol extracts have been recently used as active ingredients for active food packaging to preserve food from oxidative deterioration [147,148,149]. 

**Figure 9:**
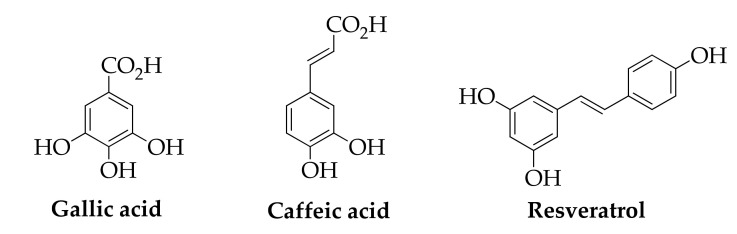
Hydroxybenzoic acids, hydroxycinnamic acids and stilbenes.

**Figure 10:**
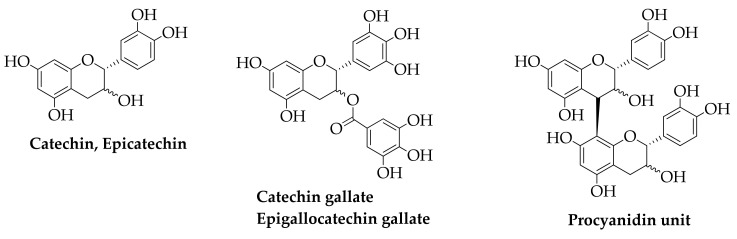
Flavan-3-ols and Proanthocyanidin unit.

The main phenolic compounds obtained from winery by-products (grape pomace, steams, leaves and seeds) are shown in Figure 9 and Figure 10. They include hydroxybenzoic acids (p-hydroxybenzoic acid, protocatechuic acid, gallic acid), hydroxycinnamic acids (p-coumaric acid, ferulic acid, caffeic acid), stilbenes (resveratrol), flavan-3-ols (catechin, epicatechin), and proanthocyanidins. These compounds show antioxidant, antimicrobial, anti-inflammatory, anticancer, and cardiovascular protection activities and are used in pharmaceutical, food, and cosmetic industries [150,151].

Proanthocyandins or condensed tannins are polymers constituted by flavonoids units; they are also found in pomegranate peel, kiwifruit peel, and are responsible for the antioxidants and anti-inflammatory activities [110,152].

**Figure 11:**
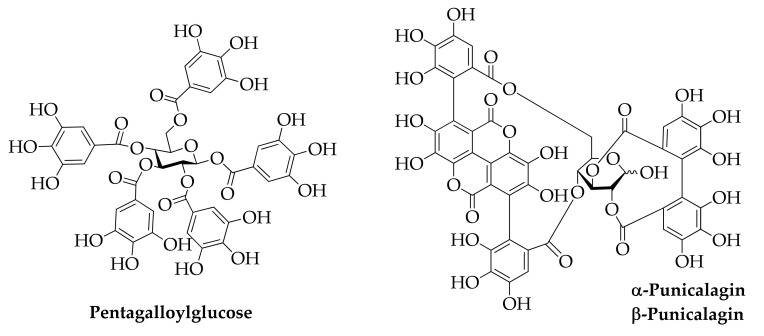
Hydrolysable tannins.

Another class of tannins are hydrolysable tannins, found in chestnut peel (Figure 11). They are polymers in which one/more molecules of gallic acid (gallotannins) or ellagic acid (ellagitannins) is/are esterified with D-glucose. Recently, they have showed antioxidant and antimicrobial activity [153,154]. For these properties, they are often used in animal feed to prevent inflammation [155].

Terpenes are a wide family of secondary metabolites found in agro-industrial wastes and by-products. They derive from the mevalonic acid biosynthetic pathway; depending on the number of the isoprene units (C5), they are classified as hemiterpenes (C5), monoterpenes (C10), sesquiterpenes (C15), diterpenes (C20), sesterterpenes (C25), triterpenes (C30), and tetraterpenes (C40). 

A high number of these compounds are toxic to insects [156], fungi, and bacteria [157]; therefore, they play an important role against insects and parasites attack. 

**Figure 12:**
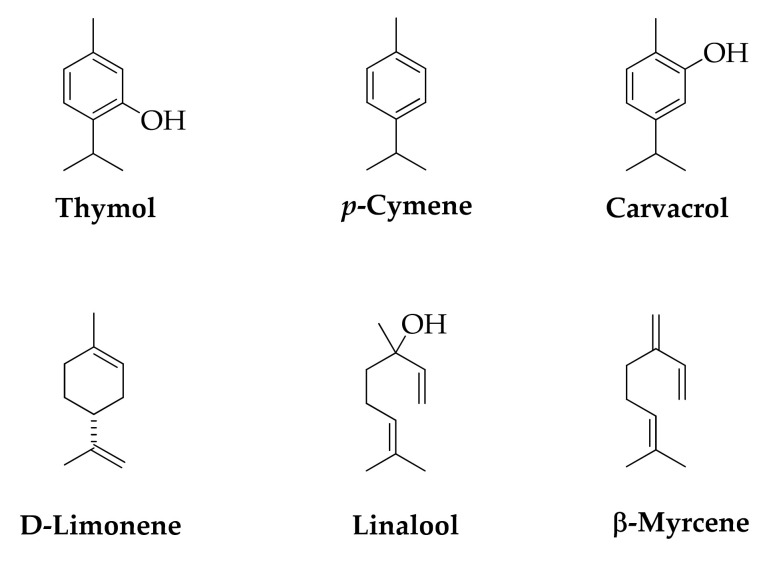
Terpenes.

Isoprene and hemiterpenes are very rare while monoterpenes are more common. Thymol, *p*-cymene, carvacrol are found in by-products derived from essential oils distillation of aromatic plants (Figure 12). These compounds are active ingredients in flavoring, perfumery, and aromatherapy [113]. 

**Figure 13:**
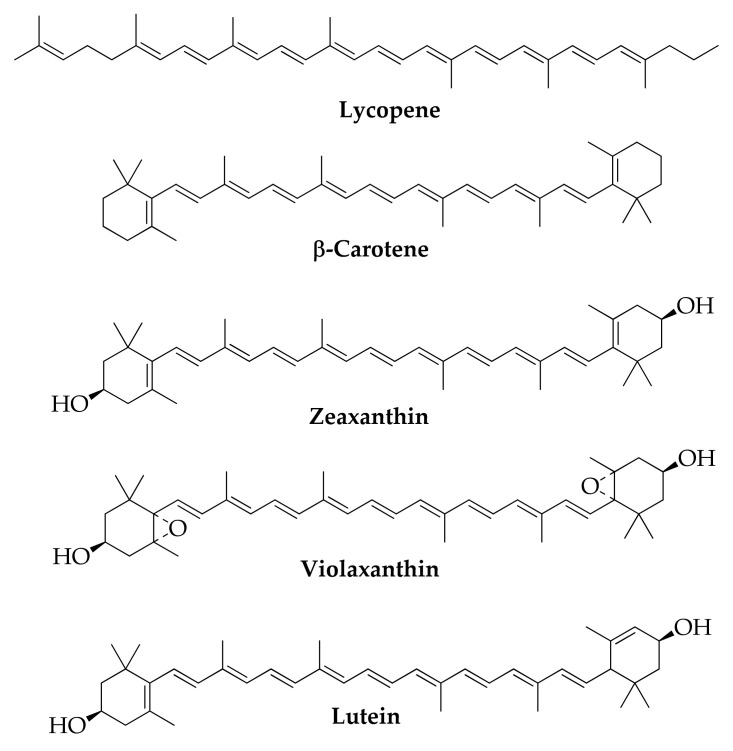
Carotenoids.

Fruit peel generated from industrial processes are sources of terpenes; in particular, orange peel waste is rich of D-limonene, linalool, and myrcene (Figure 12) [158]; tomato peel of lycopene [159] and melon peel of carotenoids as β-carotene, zeaxanthin, violaxanthin, and lutein (Figure 13) [160]. 

In industrial sectors, terpenes find several applications as biopesticides, fragrances, flavors, and food supplements [113].

## 5. Fruit Wastes and Databases

Quantification of the amount of food wastes is crucial to define reduction strategies and develop prevention campaigns over the time [161,162]. To meet these targets, the European Commission has established a common enactment for its member states to monitor food losses and waste in all steps of the food chain [163]. A challenge for food waste monitoring in member states is the development of resources, databases, and infrastructures dedicated to food waste. De Laurentiis et al. [164] estimated a household’s waste of fresh fruit and vegetables, differentiating between unavoidable and avoidable waste according to national studies conducted in six European Union (EU) member states. Caldeira et al. [165] provided a systematized approach to perform food waste accounting (food waste per product group along the food supply chain) at the EU level. It is worth mentioning the work of Joensuu et al. [166] that established a method for collecting statistical food waste data on the primary production of fruit and vegetables from horticultural producers using a questionnaire for the purposes of a national compilation of statistics on food waste. 

Besides the quantification of food waste produced, the knowledge of food waste composition has a key role in the applications and reuse of food wastes in different fields. A new goal of circular bioeconomy and biorefinery is represented by food waste as a source of bioactive compounds with a great potential in nutraceutical, cosmetic, pharmaceutical sectors [56]. Current models of existing databases, i.e., plant metabolic pathways, food composition, bioactive compounds, dietary supplements, dietary markers, and metabolomic ones, represent usable tools for health research. The need for categorization of nutrients and bioactive compounds in food and food wastes and by-products was leading to the implementation of existing databases and the development of specific and dedicated ones, based on both analytical data and collected data taken from literature throughout a harmonized and standardized approach [167]. In recent years, many studies describing the composition of nutrients and bioactive compounds were carried out as reported in previous sections.

A first effort for developing of food waste database was given by EuroFIR developing a compositional database “FoodWasteEXplorer” [168] within the EU-founded project REFRESH [169]: it provides users with ready access to the biochemical composition of agri-food chain waste streams to support research and development that can aid valorization and identification of market opportunities [170]. FoodWasteEXplorer, that encompass 27,069 data points covering 587 nutrients, 698 bioactive, and 49 toxicants, from different sources, such as scientific peer-reviewed papers, manufacturers’ data, and other sources of data. Hence, FoodWasteEXplorer can be used to investigate information on the composition of some of the most common products and their associated side streams [168,169,170].

## 6. Conclusions and Future Remarks

Fruit industry and processing results in vast quantities of biowastes, wherein FAO reported that about 30% of fruits are wasted after processing. For instance, fruit pomace, seed, oil, and cakes are rich sources of bioactive phytochemicals. Fruit biowastes are considered as a rich source of antioxidants, phenolics, pharmacologically active metabolites, dietary fibers, pigments, vitamins, and minerals, etc., which potentially have desired impact on the decrease in mortality (especially from cancers and chronic diseases), cardiovascular system, oxidative stress, metabolism, and microbial infection.

The investigation of the main components and related bioactivities of fruit wastes is being continuously explored throughout an integrated and multidisciplinary approaches towards the exploitation of emerging fields of application which may allow to create economic, environmental, and social value in an eco-friendly approach design.

The exploitation of value-added products from biomass leads to consider fruit wastes as a renewable resource in an integrated process framework that allows the extraction of all the substances of interest in a circular economy and biorefinery perspective, also in synergy with the use of innovative technologies such as nanotechnologies. The application of nanotechnologies to nutraceuticals and pharmaceuticals, offers a novel potential and novel phytochemical improvement use and opens the way to the use in the food and pharmaceutical industries [171,172,173,174]. Nowadays, several applications of nanotechnologies to the agroindustry byproducts to obtain high added value products are being carried out. For instance, Vallejo-Castillo et al. [175] proposed the development of alginate-pectin microcapsules by the extrusion for encapsulation and controlled release of polyphenols from papaya (*Carica papaya* L.) wastes. Moreover, the area of application is increasing from food and health related applications to other fields: developing edible films, biomaterials, carbon dots, microbial media, biochar and biosorbents, hydrogen production, water purification, and boiler fuel.

## Figures and Tables

**Table 1:** The top-recurring terms for fruit waste/by-product and health search (Bibliometric data were extracted from the Scopus online database and elaborated by VOSviewer software).

Term	Occurrence	Total Link Strength
fruit	38	185
fruit waste	29	216
fruits	27	277
chemistry	25	309
antioxidant	19	217
plant extract	17	247
plant extracts	17	237
antioxidants	17	185
nonhuman	17	164
bioactive compounds	16	113
humans	13	171
human	12	163
antioxidant activity	11	124
metabolism	10	101
food processing	10	79
fermentation	10	75
plant seed	9	129
phenols	8	83
waste disposal	8	40
phytochemicals	7	113

**Table 2:** Main fruit waste and by-products available in the scientific literature and/or exploited in industrial processes.

Fruit	Waste/By-Products	Biochemicals/Biomaterials Exploitable	Extraction Techniques Reported	References
apple	peel pomace	dietary fiber pectins polyphenols	enzyme-assisted extraction, microwave-assisted extraction, pressurized liquids extraction, pressurised liquid extraction	[59,66,67,85,90,101]
banana	peel	pectins	enzyme-assisted extraction	[83]
berries	blueberry fruits blueberry pressed cake raspberry waste	anthocyanins polyphenols	enzyme-assisted extraction, pulsed electric field extraction	[64,100]
citrus fruits	bergamot albedo grapefruit peel lemon peel lime peel mandarin peel orange peel	carotenoids essential oils flavonoids naringin pectins pigments carotenoids polyphenols	enzyme-assisted extraction, microwave-assisted extraction, ultrasound-assisted extraction, microwave-assisted hydrodistillation, supercritical fluid extraction, pressurised liquid extraction	[49,65,80,81,87,88,89,97,98,107]
grape	pomace peel seeds	aroma compounds anthocyanins flavonoids organic acids polyphenols pectins polysaccharides procyanidins procyanidin dimers A and B	enzyme-assisted extraction, microwave-assisted extraction, ultrasound-assisted extraction, supercritical fluid extraction, pressurised liquid extraction	[56,60,61,62,71,76,94,95,99,102,106,108,109]
jackfruit	peel	pectins	microwave-assisted extraction, ultrasound-assisted extraction	[96]
kiwifruit	peel thinning waste	pectins polyphenols	enzyme-assisted extraction, pressurised liquid extraction	[68,110]
mango	peel	pectins polyphenols	ultrasound-assisted extraction	[72,86,93]
papaya	peel	dietary fiber	ultrasound-assisted extraction	[85]
		pectins		
passion fruit	peel	pectins polyphenols	enzyme-assisted extraction, ultrasound-assisted extraction, pressurised liquid extraction, supercritical fluid extraction	[18,69,78]
pomegranate	peel	pectins polyphenols	enzyme-assisted-supercritical fluid extraction, enzyme-assisted extraction, ultrasound-assisted extraction, pressurised liquid extraction	[70,72,77]
tropical fruits	mango mango leaves passion fruit seeds passion fruit cake	alkaloids essential oils flavonoids naringenin phenolics terpenic compounds	enzyme-assisted extraction, supercritical fluid extraction, pressurised liquid extraction	[103,111,112]

## Data Availability

Not applicable.
